# Fungous Tumor of the Fangs of a Molar Tooth, Diagnosed and Treated as a Case of Menoplania

**Published:** 1869-07

**Authors:** Geo. Syng Bryant

**Affiliations:** Lexington, Ky.


### ARTICLE IX.

### *Fungous Tumor of the Fangs of a Molar Tooth, Diagnosed and Treated as a case of Menoplania.*

By Geo. Syng Bryant, M.D., Lexington, Ky.

Miss—, æt. 16 years ; a blonde ; tall, thin and pale ; skin of a chlorotic cast. Has suffered much from palpitation of the heart and indigestion ; appetite has usually been depraved for several months past.

Prior to the last eighteen months this young lady had been healthy and ruddy ; but being ambitious to outstrip her classmates she gave herself up to arduous study and soon her health began to fail. She did not, however, relax her efforts at the school-form. Periodical headache, attended sometimes with sick stomach, would compel her to leave the school-room and retire to bed ; these attacks lasting from 24 to 48 hours.

At fourteen years of age the catamenial eruption took place and continued regular and normal for about nine months; then became irregular and scanty, and finally ceased altogether. About the time of the cessation of the catamenia the headaches grew worse and more frequent and were usually attended with an uneasy sensation, or slight aching of the second lower molar tooth of the left side. After a few weeks, the head symptoms and toothache became so much worse that continuance at school was impossible.

The tooth being apparently sound, the physician in attendance, regarding both the tooth and headache of neuralgic character, and dependent upon close confinement and hard study, put the patient upon tonics, and advised exercise in the open air whenever practicable. It was observed that the bleeding from the gums of the affected tooth was assuming a periodical tendency. The physician believed this hæmorrhage to be an example of vicarious menstruation, and the case attracted much attention. Being called in consultation, I learned the above history, substantially, from the family physician. It was evident, to my mind, that the young lady was laboring under a chlorotic amenorrhœa, with great impairment of the nutritive functions, which, in this case, was probably the primary etiological element of the chlorosis and amenorrhœa. Upon close inspection of the affected tooth, I discovered some enlargement, and slight inflammation of the gums around it. Being fully persuaded that this was a case of disease of the fangs of the tooth, accompanied with periodical hæmorrhage, and that it was not a case of menoplania, I advised that the tooth be extracted immediately, and the patient put upon treatment suited to her amenorrhœal and chlorotic condition. This opinion was opposed by the attending physician and another present in the consultation. Upon being sent for again, in in the course of two weeks, I urged the pulling of the tooth which was consented to, and on the following day the tooth was extracted by a skillful dentist. Both fangs were found affected with a fungous tumor—one the size of a small pea, and the other not as large. I need scarcely add that the hæmorrhage never returned, the headaches grew less frequent and severe, and in a few months the attenuated, nervous young sufferer was fully restored to health.

May not many of the so-called examples of vicarious menstruation be only cases of amenorrhœa, complicated by other diseases.—*Richmond and Louisville Med. Jouraal.*

